# Transient acoustic vaporization signatures unique to low boiling point phase change contrast agents enable super-resolution ultrasound imaging without spatiotemporal filtering

**DOI:** 10.1063/5.0029207

**Published:** 2020-10-19

**Authors:** R. M. DeRuiter, E. N. Markley, J. D. Rojas, G. F. Pinton, P. A. Dayton

**Affiliations:** Joint Department of Biomedical Engineering, University of North Carolina–Chapel Hill and North Carolina State University, 116 Manning Drive, 9018 Mary Ellen Jones Building, CB7575, Chapel Hill, North Carolina 27599, USA

## Abstract

The unique activation signal of phase-change contrast agents (PCCAs or droplets) can be separated from the tissue signal and localized to generate super-resolution (SR) ultrasound (US) images. Lipid-shelled, perfluorocarbon PCCAs can be stochastically vaporized (activated) by a plane wave US transmission thereby enabling them to be used as separable targets for ultrasound localization microscopy. The unique signature of droplet vaporization imaging and the transient inherent nature of this signature increases signal contrast and therefore localization confidence, while the poor resolution of the low-frequency vaporization signal is overcome by the super-resolution result. Furthermore, our proposed PCCA SR technique does not require the use of user-dependent and flow-dependent spatio-temporal filtering via singular-value decomposition. Rather, matched filters selected by Fourier-domain analysis are able to identify and localize PCCA activations. Droplet SR was demonstrated in a crossed-microtube water phantom by localizing the activation signals of octafluoropropane nanodroplets (OFP, C_3_F_8_, −37 °C boiling point) to resolve 100 *µ*m diameter fluorinated ethylene propylene tubes, which are ordinarily 35% smaller than the native diffraction-limited resolution of the imaging system utilized.

Phase change contrast agents (PCCAs) are a class of ultrasound (US) contrast agents whose core substance changes the state of matter during use. This is unlike conventional microbubble contrast agents (MCAs), which have gaseous cores that do not require a phase change. The shell that encapsulates many PCCA formulations increases the stability of the liquid (droplet) phase, which enables the PCCA to remain in a superheated liquid state at room temperature and for *in vivo* applications. Acoustic droplet vaporization (ADV), also known as activation, can be achieved via ultrasonic energy and other energy inputs, such as thermally or optically.^1–4^ Early PCCAs, particularly in the case of sub-micron droplets, required high acoustic energy to activate, which raised the risks of undesired bioeffects. Low-boiling point PCCAs were introduced to address these concerns.^5^ Decafluorobutane (DFB, C_4_F_10_, −2 °C boiling point) and octafluoropropane (OFP, C_3_F_8_, −37 °C boiling point) are both gaseous at room temperature and, when condensed, form superheated droplets on the order of hundreds of nanometers in diameter. Vaporization of these nanodroplet PCCAs requires less acoustic energy than traditional perfluorohexane or perfluoropentane PCCAs.^6^ *In vivo* assessment of low-boiling point PCCA activation in the kidney has demonstrated that vaporization is possible without bio-effects using moderate (0.81–1.35) mechanical indices (MIs).^7^

Low-boiling point perfluorocarbon-based PCCAs are of interest for contrast imaging due to the unique ultrasound signal produced by activation.^6^ Measurements of single droplet activation showing the characteristic decaying sinusoidal signal are provided in Fig. 1. The resulting activation signal is determined by the PCCAs size, stability, and sensitivity to US, but its frequency content can generally be characterized as low frequency (100 kHz to 4 MHz for resulting microbubble sizes of 11 *µ*m–1 *µ*m, respectively).^6^ This unique signal has been the target of several other PCCA-specific imaging techniques, such as dual frequency vaporization detection^8^ and vaporization detection imaging (VDI).^9^ In both detection methods, the dual frequency scheme employed a high frequency transmit bandwidth (BW) and a low frequency receive BW. This imaging scheme allows the PCCAs to be activated at the high frequency and importantly allows the characteristically low frequency of the resulting activation signals to be detected at this low frequency with limited non-contrast signal from the transmission. Consequently, these dual-frequency schemes can yield high contrast-to-tissue ratios (CTRs). In effect, the lower frequency PCCA activation signals could be distinguished from tissue and other signals by transmitting with a spectrally isolated center frequency (in practice, a higher frequency transmission). Ideally, spectral means alone could therefore be used to discriminate the low-frequency response of the contrast from the high frequency linear response of the background. Even in the non-ideal case where the BWs of the transmission and the resonance of the activation signal overlap, the activation signal is a unique feature that can be isolated by matched filtering. Acoustic angiography, another dual frequency contrast US imaging technique, discriminates the contrast signal by the opposite scheme: a low-frequency transmit BW with a high-frequency receive BW that solely captures superharmonic content.^10^

**FIG. 1.**
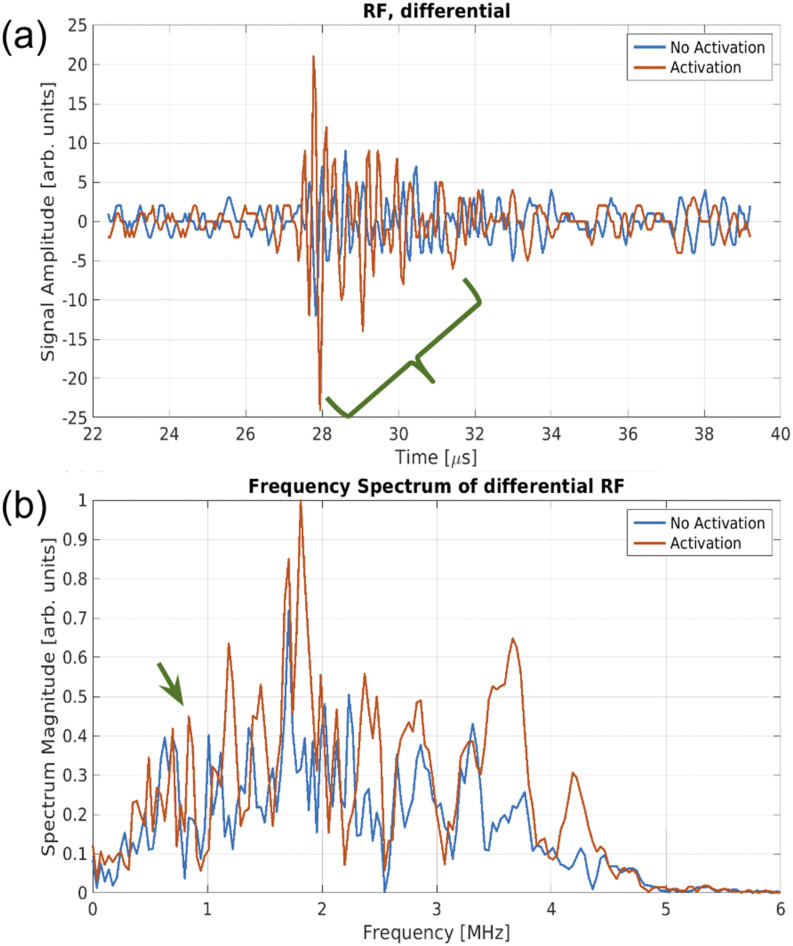
Experimental single-frame differential signal measurement of octafluoropropane droplets vaporizing (a) and the corresponding frequency spectra (b). The green brace in (a) denotes the activation signal, while the green arrow in (b) represents the frequency peak represented by the decaying peaks of the vaporization signal. The difference between the activation signal vs non-activating nanodroplet response to US is apparent in the red vs blue plots of (a), but less apparent in (b) alone.

Super resolution (SR) images of vasculature can be generated by ultrasound localization microscopy (ULM), the acoustic analog to optical fluorescence photoactivation localization microscopy (FPALM)^11^ and stochastic optical reconstruction microscopy (STORM).^12^ By these processing techniques, separable signals from microbubble contrast agents can be accurately localized and accumulated to surpass the typical diffraction limit of the imaging system. Acoustic wave sparsely activated localization microscopy (AWSALM) is a technique by which PCCAs are first activated, and the resulting MBs are then imaged and subsequently destroyed.^13^ This first version utilized focused transmit pulses to activate DFB PCCAs and had a separate imaging pulse sequence. Even more recently, an updated version, fast-AWSALM, was published that uses plane wave imaging to activate OFP PCCAs.^14^ Similar to AWSALM, the localization target of fast-AWSALM for ULM is still the resulting microbubbles, post-activation. Unlike AWSALM, the research presented here instead looks to localize the activation signals of the PCCAs themselves. Both AWSALM methods utilize the singular value decomposition (SVD) method of spatio-temporal filtering to help differentiate contrast agent signal from the background. Filtering via SVD provides limitations of detection during slow flow.^15^ The method we propose here does not rely on spatiotemporal filtering, which can provide an advantage when imaging slow flow.^15^ The SVD representation of a dataset of images is peculiar to that dataset, and filter parameters are often not robust between datasets. The success of the filtering can consequently be user-dependent.

In this research, we exploit the transient and unique acoustic signature of dual frequency droplet activation imaging^8,9^ to generate SR vascular images. Provided that the activations are separable, they can be accurately localized and accumulated with similar processing techniques as ULM, one of the primary methods of achieving SR images. ULM imaging can generally be described by four steps: data acquisition, signal discrimination, localization, and accumulation.

Methods of data acquisition vary in the literature. Plane wave transmission schemes can be used to achieve very high frame rates as in the case of ultrafast ULM (uULM),^16^ while more complex imaging schemes such as pulse-inversion (PI) can be used to improve signal contrast at the expense of frame rate.^17^ Using an ATL L11-5 linear array controlled by a Vantage 256 ultrasound research platform (Verasonics, Kirkland, WA, USA), we transmit 5 MHz, one cycle plane waves at 510 kPa (0.22 MI) at a frame rate of 200 Hz. The same linear array was used for transmit and receive. Contrary to previous high-frequency transmit BW and low-frequency receive BW imaging schemes described previously,^8,9^ additional low-pass filtering was not conducted on the raw radio-frequency data. This omission was necessary to capture activation signals that manifested at higher than expected frequencies (∼4 MHz). As an imaging target, we used two 100 *µ*m inner-diameter fluorinated ethylene propylene (FEP) capillary tubes (Paradigm Optics, now INCOM Inc., Charlton, MA, USA) within a water phantom, which would otherwise be below the typical resolution limit for a one cycle, 5 MHz imaging scheme (Fig. 2). The water temperature was maintained at 37 °C by an ISOTEMP 2013S recirculating water bath (Fisher Scientific, Waltham, MA, USA) and monitored in real-time by using a Fluke 51 II digital thermometer (Fluke Corporation, Everett, WA, USA).

**FIG. 2.**
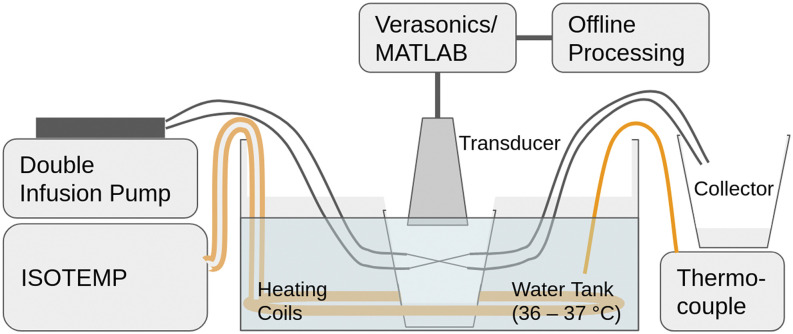
Full experimental setup. The heating coils kept the water in the water tank and in the water phantom at 37 °C, as read by the thermocouple. The double infusion pump supplied the PCCAs through the tube at a constant infusion rate and into a collector. The imaging transducer was positioned downwards such that the tubes were crossing in-plane.

The PCCAs used consisted of a lipid shell and an OFP core, formulated as previously described^18^ and detailed in Subsection A of the supplementary material. Characterization of the PCCAs including size distribution can similarly be found in Subsection B of the supplementary material. Once diluted to a concentration of 1 × 10^9^ No./ml, the PCCAs were then infused through the microtube at a controlled rate of 50 *µ*l/min by a PHD 2000 programmable dual infusion syringe pump (Harvard Apparatus, Holliston, MA, USA). In this imaging scheme, the single plane wave transmission provided both the imaging pulse and the activation pulse, as the OFP PCCAs were selected for their heightened volatility. Although not demonstrated fully by our 200 Hz frame rate, this fact should enable this imaging scheme to be conducted at higher frame rates to limit acquisition times providing sufficient concentration or reperfusion of PCCAs for stochastic activation. The raw RF data were saved for offline reconstruction and processing.

The next step in ULM after data acquisition is signal discrimination. Ultimately, signal contrast in the raw data/image determines what is necessary in the signal discrimination step. For uULM, this discrimination often comes from spatio-temporal filtering in the form of removing select eigenbases of the SVD of the image data. In theory, this filtering should remove eigenbases that represent background (tissue) and noise while preserving eigenbases that represent the contrast agent. In practice, however, the discrimination between desired and undesired signals is imperfect as spatiotemporal eigenbases are difficult to interpret. Notably, it is difficult to distinguish the slow-flowing contrast signal from the stationary background signal, making small vessels with inherently slow flow difficult to resolve. This difficulty of interpretation can often be further complicated by tissue motion, which interferes with faster moving flow on the same order of magnitude as the tissue motion. In our case, the differentiation of PCCA activation vs background is provided by spectral filtering, so SVD is not necessary for our proposed technique. It is important to note the bandwidth (BW) of the transducer used for imaging. Empirical characterization of our specific L11-5 probe is provided in Subsection C of the supplementary material. In short, the L11-5 probe is not ideal to detect low frequency signals that are outside of its supported BW (5 MHz–11 MHz, −3 dB). For example, activation signals detected on the lower extreme of the BW at 4 MHz in frequency corresponded to the activation of 200 nm diameter PCCAs, based upon theoretical modeling derived from the classic Minnaert equation for the microbubble resonant frequency.^6^ The activation of these 200 nm PCCAs would result in microbubbles of diameters ∼1 *µ*m. To improve the contrast and to remove any non-activation artifacts that may have been detected, a frame-by-frame subtraction was implemented on the dataset, similar to differential imaging (DI) in the literature.^19^ This single frame differential imaging is possible due to the transient nature of the activation signal. The PCCA activation signal and resulting microbubble (MB) oscillations occur on the order of microseconds,^20^ and because the imaging pulse and activation pulse are necessarily synchronous in our single pulse imaging scheme, the activation signal of any given PCCA is constrained to appear only in the frame reconstruction in which it was activated.

Once the PCCA signal is identified, the coordinates of the signal’s source are then determined for a high-resolution grid in the third ULM step: localization. Necessarily, in order to resolve two contrast signals from one another, the two sources must be separable. Our localization methods are designed to exploit the ringing effect of the PCCA activation signal. In short, the frequency content of suspected activation signals was first analyzed and then localized by a normalized cross correlation with an appropriate matched filter selected to exploit the characteristic ringing signal of the activation. Although PCCAs of different sizes have slightly variable frequency content with respect to one another, the localization methods employed here are robust enough to automatically select a matched filter suitable for the detected frequency. PCCA activation localization is provided in greater detail and with visuals in Subsection D of the supplementary material. Not only does this matched-filter-based approach provide the localization of the activation signal, it also provides a necessary contrast improvement vs noise, as the noise is very unlikely to pass through the filter.

Finally, the SR image itself can be generated by accumulating the localization counts across the acquired frames. The super-resolved result of our PCCA activation-localization SR technique is presented in Fig. 3(b). A profile measurement of the crossed tubes at a distance where the two are less than half a wavelength apart is provided in Fig. 3(c) and is compared against the profile resolution of a maximum intensity projection (MIP) of the B-mode images through slow-time at the transmit frequency of 5 MHz [Fig. 3(a)]. The full-width at half-maximum (FWHM) measurements of the average cross section across the length of each tube were 81 *μ*m and 91 *µ*m, which are somewhat smaller than the expected value of 100 *µ*m.

**FIG. 3.**
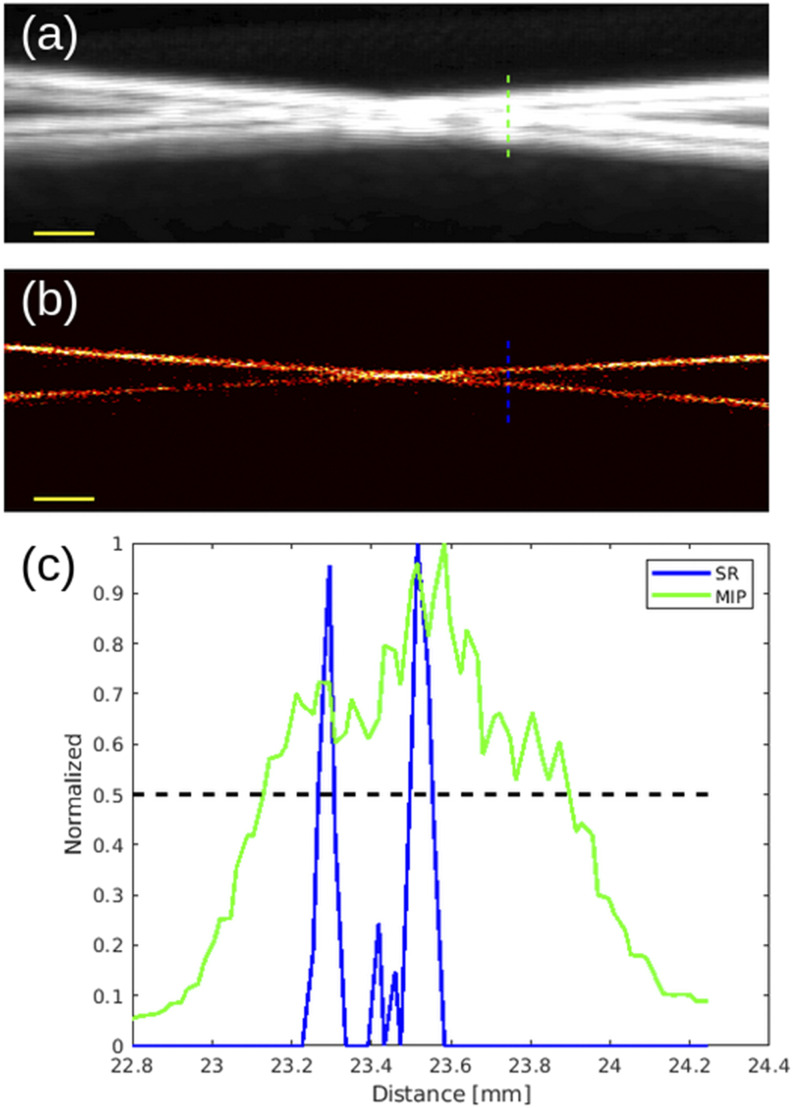
(a) Maximum intensity projection (MIP) of activation signals through frames. (b) Resultant super-resolution (SR) image from phase-change contrast agent activation localization. (c) Profile measurements taken at the dashed lines indicated in (a) and (b): MIP profile in green and SR profile in blue. The full-width at half-maximum (FWHM) location is provided. Scale bars (yellow) for (a) and (b) and are 1 mm.

This result demonstrates the feasibility of the localizing activation signal of PCCAs for SR imaging. The poor resolution inherent to droplet activation imaging was overcome by the SR imaging techniques to resolve sub-resolution tubes *in vitro*. However, there are a few key considerations as this technique moves toward *in vivo* translation. First, the attenuating properties of biological tissue may present a barrier to insonifying the PCCAs with enough acoustic energy for vaporization in deep imaging cases. One of two possible solutions may be utilized in this case. Either the plane wave transmission will need to be focused to that of a wide-beam focused scheme or the typically polydisperse PCCAs will need to be size-selected in favor of larger PCCAs, which would be most likely to activate at lower pressures. In the case of focused ultrasound, the attenuation paths of the ultrasound will have to be considered in order to ensure that the correct pressures for acoustic vaporization can be controlled. This control could be achieved by using an activation pressure matching method.^21^ In addition, the trade-off between frame-rate and perfusion must be considered. Since OFP droplets do not recondense post-activation, each individual PCCA can only be imaged by this technique one time. Consequently, it becomes important that there are sufficient PCCAs within the imaging target that stochastic activation is sustainable over the imaging period. Thus, the imaging frame-rate should be selected with the perfusion of PCCAs in mind. Interestingly, because the single-frame transience of the activation signal is what provides the contrast in this SR imaging technique, flow is not required to detect the contrast, as is the case with the SVD and other spatio-temporal-based methods. This could open PCCA activation-based localization to applications where flow is absent, as would be the case for molecular imaging, where targeted contrast agents bind to physiological receptors. This research does not investigate the effects of different flow rates on the accuracy of the localization processing nor does it determine a theoretical resolution limit of the proposed imaging technique. While it can be hypothesized that the effect of flow rate on the appearance of the resultant PCCA activation signal, and therefore its ability to be robustly localized, is likely small relative to the effects of the PCCA activation oscillation frequency and the transience of the signal itself, this could be the subject of future efforts. Additionally, the selection of imaging transducer should be reexamined. As mentioned, the L11-5 used in this research did not allow for a high-frequency transmit, low-frequency receive PCCA-specific imaging scheme, thereby lowering the achievable contrast-to-noise ratio (CNR). Another transducer with greater sensitivity at lower frequencies would likely have more obvious CNR advantages vs traditional US contrast imaging. For processing, improvements could be made in the localization processing step by applying the matched filtering on the raw RF data. The matched filtering at this stage could be more specific to the activation signal than it is at the beamformed stage, thereby offering greater contrast signal overall for localization. The matched filtering alternative can be visualized in Subsection E of the supplementary material. Finally, more future research studies could provide a comprehensive comparison of this proposed technique with the existing methods for SR imaging, such as SVD filtering and differential imaging of conventional microbubble contrast agents.

In summary, this research presents a novel imaging technique to create ultrasound super-resolution images by localizing the unique activation signals of low-boiling point phase-change contrast agents.

See the supplementary material for details regarding PCCA fabrication and characterization, transducer characterization, and additional processing specifics, including the current approach for PCCA activation localization steps and the potential approach for localization in RF.

## Data Availability

The data that support the findings of this study are available from Ryan DeRuiter upon reasonable request: enry03@email.unc.edu.
